# Identification and molecular characterization of Taro bacilliform virus and Taro bacilliform CH virus from East Africa

**DOI:** 10.1111/ppa.12921

**Published:** 2018-08-31

**Authors:** D. B. Kidanemariam, A. C. Sukal, A. D. Abraham, F. Stomeo, J. L. Dale, A. P. James, R. M. Harding

**Affiliations:** a Centre for Tropical Crops and Biocommodities, Queensland University of Technology, Brisbane 4001, Australia; b National Agricultural Biotechnology Research Centre, Ethiopian Institute of Agricultural Research, PO Box 2003; c Department of Biotechnology, Addis Ababa Science and Technology University, PO Box 16417, Addis Ababa, Ethiopia; a Biosciences eastern and central Africa-International Livestock Research Institute (BecA-ILRI) Hub, PO Box 30709, Nairobi, Kenya

**Keywords:** badnavirus, *Caulimoviridae*, *Colocasia esculenta*, episomal DNA, rolling circle amplification, *Xanthosoma*

## Abstract

Taro (*Colocasia esculenta*) and tannia (*Xanthosoma* sp.) are important root crops cultivated mainly by small-scale farmers in sub-Saharan Africa and the South Pacific. Viruses are known to be one of the most important constraints to production, with infections resulting in severe yield reduction. In 2014 and 2015, surveys were conducted in Ethiopia, Kenya, Tanzania and Uganda to determine the identity of viruses infecting taro in East Africa. Screening of 392 samples collected from the region using degenerate badnavirus primers revealed an incidence of 58–74% among the four countries surveyed, with sequence analysis identifying both *Taro bacilliform virus* (TaBV) and *Taro bacilliform CH virus* (TaBCHV). TaBCHV was identified from all four countries while TaBV was identified in all except Ethiopia. Full-length sequences from representative TaBV and TaBCHV isolates showed that the genome organization of TaBV isolates from East Africa was consistent with previous reports while TaBCHV isolates from East Africa were found to encode only four ORFs, distinct from a previous report from China. Phylogenetic analysis showed that all East African TaBV isolates form a single subgroup within known TaBV isolates, while TaBCHV isolates form at least two distinct subgroups. To the authors’ knowledge, this is the first report describing the occurrence and genome organization of TaBV and TaBCHV isolates from East Africa and the first full-length sequence of the two viruses from tannia.

## Introduction

The aroids taro (*Colocasia esculenta*) and tannia (*Xanthosoma* sp.) are among the most important root crops in many sub-Saharan African countries (Ndabikunze *et al*., 2011; Akwee *et al*., 2015). The corm of taro and tannia plants are sources of starch and dietary fibre and also contain substantial amounts of protein, vitamins and minerals (Ndabikunze *et al*., 2011; Akwee *et al*., 2015). In East African countries, both these aroids are mainly cultivated by smallholder farmers where they play important cultural, economic and nutritional roles (Onwueme & Charles, 1994; Talwana *et al*., 2009; Tumuhimbise *et al*., 2009; Beyene, 2013).

Badnaviruses have bacilliform-shaped particles of approximately 30 nm by 120–150 nm with a circular, double-stranded (ds) DNA genome of 6.9–9.2 kb. The genome typically contains three open reading frames (ORFs) but there may be one or more additional ORFs (Geering & Hull, 2012; Bhat *et al*., 2016). ORFs 1 and 2 encode small proteins of about 23 and 15 kDa, respectively (Geering & Hull, 2012). The function of the protein encoded by ORF 1 is unknown, while the ORF 2 protein has nonspecific DNA- and RNA-binding activity and may be involved in virion assembly (Jacquot *et al*., 1996). ORF 3 encodes a large polyprotein of *c*. 200 kDa that is post-translationally processed into several mature proteins, including movement protein (MP), coat protein (CP), aspartic protease (AP), reverse transcriptase (RT) and ribonuclease H (RNase H) (Geering & Hull, 2012; Bhat *et al*., 2016). Several additional ORFs have been reported from a number of species; however, these usually have no ascribed function (Kazmi *et al*., 2015). The RT/RNase H-coding region of ORF 3 is the most conserved region of the genome and a nucleotide (nt) difference of greater than 20% in this part of the genome is used for the demarcation of species in the genus (Geering & Hull, 2012).

The genus *Badnavirus* is the most diverse member of the family *Caulimoviridae* at both the genomic and antigenic level and currently comprises 40 distinct recognized species (Geering & Hull, 2012; https://talk.ictvonline.org/taxonomy/). In taro, two distinct badnaviruses have been reported, namely *Taro bacilliform virus* (TaBV; Yang *et al*., 2003a,b) and *Taro bacilliform CH virus* (TaBCHV; Ming *et al*., 2013; Kazmi *et al*., 2015). The genome of TaBV possesses four ORFs, all encoded on the plus-strand of the viral DNA, with the size and organization of ORFs 1–3 consistent with most badnaviruses (Yang *et al*., 2003a). ORF 4 of TaBV overlaps ORF 3 between the MP and CP domains and putatively encodes a protein of c. 13 kDa, with little homology to any published protein-coding sequences (Yang *et al*., 2003a). In contrast to TaBV, TaBCHV encodes six putative ORFs, with ORFs 1–4 analogous to TaBV and an additional two small ORFs at the 3′ end of ORF 3. ORF 5 partially overlaps ORF 3, while ORF 6 is downstream of, and partially overlaps, the 3′ end of ORF 5 (Kazmi *et al*., 2015). Characterization of Pacific isolates of TaBV showed that there is up to 23% nucleotide sequence variability within the RT/RNase H-coding region (Yang *et al*., 2003b). The same study also revealed the presence of TaBV-like sequences in taro samples from Papua New Guinea (PNG), Fiji, Vanuatu, Samoa, Solomon Islands and New Caledonia with 50–60% nucleotide identity to TaBV, indicating the possible presence of other badnaviruses infecting taro in the South Pacific region. Recently, TaBCHV has been reported from Hawaii (USA), with 91–98% nucleotide sequence identity to the published TaBCHV isolate from China (Wang *et al*., 2018).

To date, TaBV and TaBCHV appear to be restricted to host plants in the family Araceae. TaBV is transmitted mainly by vegetative propagation, mealybugs in a semipersistent manner, and in some cases through seed or pollen, but it is not mechanically transmissible (Gollifer *et al*., 1977; Macanawai *et al*., 2005). Although no consistent symptoms have been associated with TaBV infection, there have been some reports of mild symptoms such as vein clearing, stunting and downward curling of the leaf blades in some cultivars (Yang *et al*., 2003a; Revill *et al*., 2005; Kidanemariam *et al*., 2018).

Despite the importance of aroids in sub-Saharan Africa, there is currently no information on the incidence, distribution and diversity of TaBV or TaBCHV in the region. In 2014 and 2015, surveys were conducted to identify viruses infecting taro and other edible aroids in Ethiopia, Kenya, Tanzania and Uganda. This paper reports the identification and genomic characterization of both TaBV and TaBCHV from East African countries and discusses their incidence and sequence diversity.

## Materials and methods

### Sample collection and DNA extraction

Between November 2014 and August 2015, leaf samples were collected from 333 taro plants and 59 tannia plants from 25 major growing areas in Ethiopia, Kenya, Tanzania and Uganda. Of these, 171 (160 taro and 11 tannia) were collected from Ethiopia, 86 (83 taro and three tannia) from Kenya, 41 (29 taro and 12 tannia) from Tanzania and 94 (61 taro and 33 tannia) from Uganda (Table 1). Samples were taken from plants showing virus-like symptoms as well as from symptomless plants. The leaf samples were desiccated over silica gel and transported to the BecA-ILRI hub laboratory in Nairobi, Kenya for *in vitro* laboratory analysis. Total nucleic acid (TNA) was extracted using 2% CTAB as described by Kleinow *et al*. (2009). Selected nucleic acid samples were later transported to Queensland University of Technology (QUT), Brisbane, Australia for cloning and sequence analysis.

**Table 1:** Location of taro and tannia samples collected in this study, and summary of initial PCR testing Country District

Country	District	Number of samples	Taro		Tannia		Total		Samples selected for sequencing
			Total	Number positive	Total	Number positive	Number positive	% positive	
Ethiopia	Welayita	87	84	75	3	1	76	87.4	Et4, Et8, Et17, Et22, Et141, Et158
	Oromia	22	22	1	0	0	1	4.5	Et72
	Sheka	25	22	7	3	3	10	40.0	Et43
	Masha	14	12	3	2	1	4	28.6	Et49
	Kefa	23	20	6	3	3	9	39.1	Et50
	Total	171	160	92	11	8			10
Kenya	Nyeri	30	29	17	1	0	17	56.7	Ke65, Ke72
	Laikipia	3	2	1	1	0	1	33.3	
	Tharaka Nithi	14	14	10	0	0	10	71.4	Ke14, Ke16, Ke18
	Kirinyaga	9	8	5	1	0	5	55.6	
	Embu	19	19	13	0	0	13	68.4	Ke43, Ke49, Ke51, Ke52
	Kakamega	4	4	4	0	0	4	100.0	
	Kisumu	5	5	3	0	0	3	60.0	Ke83
	Siaya	2	2	1	0	0	1	50.0	
	Total	86	83	54	3	0			10
Tanzania	Musoma	9	9	3	0	0	3	33.3	Tz7
	Tarime	5	2	1	3	3	4	80.0	
	Mago	2	2	2	0	0	2	100.0	Tz16, Tz17
	Biharamulo	9	1	0	8	8	8	88.9	Tz24^a^, Tz27^a^
	Mwanza	16	15	7	1	1	8	50.0	Tz36, Tz42, Tz43, Tz44, Tz47
	Total	41	29	21	12	4			10
Uganda	Busuju	25	16	15	9	4	19	76.0	Ug6, Ug10, Ug15
	Lukaaya	26	17	15	9	5	20	76.9	Ug35, Ug45, Ug52
	Busiro	20	11	10	9	1	11	55.0	Ug67
	Budondo	4	4	4	0	0	4	100.0	Ug75
	Buunya	6	5	5	1	0	5	83.3	Ug79
	Kignlu	3	2	2	1	1	3	100.0	
	Luuka	10	6	5	4	3	8	80.0	Ug96
	Total	94	61	56	33	14			10

a Tannia sample sequenced.

### PCR, cloning and sequencing

PCR was carried out using OneTaq 2× master mix (NEB) and degenerate badnavirus primers BadnaFP/RP as described by Yang *et al*. (2003a), and amplicons were separated by electrophoresis through 1.5% agarose gels. As a positive control, total DNA extracted from yam leaf tissue infected with *Dioscorea bacilliform alata virus* was used.

Ten PCR positive samples from each country, representing different districts where possible, were randomly selected and amplicons of the expected size (*c*. 580 bp) were gel-excised and purified using the Freeze ‘N’ Squeeze DNA Gel Extraction Spin Columns (Bio-Rad) and subsequently cloned into pGEM-T Easy (Promega). Putative recombinant plasmid DNA containing the PCR amplicons was sequenced using the Big Dye Terminator v. 3.1 Cycle Sequencing kit (Thermo Fisher Scientific) at the Central Analytical Research Facility (CARF), QUT, Brisbane, Australia. For each sample, three independent clones were sequenced in one direction using the M13F primer.

### Rolling circle amplification (RCA), restriction digestion, cloning and sequencing

RCA was carried out using the Illustra TempliPhi 100 Amplification kit (GE Healthcare) as described by James *et al*. (2011). The RCA products were digested with *Stu*I, *Sal*I and *Xba*I restriction enzymes (NEB). *In silico* restriction site analysis based on published full-length sequences of TaBV (Yang *et al*., 2003a; GenBank accession no. AF357836) and TaBCHV (Kazmi *et al*., 2015; GenBank accession no. NC026819) predicted that these enzymes would cut up to three times. Digested RCA products were separated using 0.8% agarose gels and fragments of *c*. 7–8 kb were excised, purified as described previously and subsequently ligated into appropriately digested and alkaline phosphatase-treated pUC19 plasmid DNA. Full-length genome sequences were subsequently generated from putative recombinant plasmid DNA containing the RCA-derived amplicons, with sequencing carried out as described previously. For each sample, at least three independent clones were sequenced in both directions. To confirm the sequences spanning the putative restriction sites, PCR was carried out using sequence-specific primers flanking the region. Briefly, PCR master mix consisted of 10 μL of 2× GoTaq Green master mix (Promega), 5 pmol of each sequence-specific primer and 1 μL of TNA (30 ng μL^−1^) in a final volume of 20 μL. PCR cycling conditions were as follows: initial denaturation at 94 °C for 3 min; 35 cycles of 94 °C for 30 s, 50 °C for 30 s, and 72 °C for 2 min; and a final extension at 72 °C for 10 min. The amplified products were cloned into pGEM-T Easy and sequenced as described previously.

### Outward-facing PCR

To amplify the complete genome sequence of TaBCHV isolates from East Africa, outward-facing, sequence-specific primers (TaBCVH-OutF: 5′-AGGCCCATTATACTCAAAAG-3′ and TaBCHV-OutR: 5′-GAAATCAATGGTTGGTACTG-3′) were designed based on consensus RT/RNase H-coding sequences obtained in this study. Long-range PCRs were carried out using 1 μL of TNA (30 ng μL^−1^) mixed with 10 μL of 2× GoTaq long-range PCR master mix (Promega) and 5 pmol of each sequence-specific primer in a final volume of 20 μL. PCR cycling was as follows: initial denaturation at 94 °C for 3 min; 30 cycles of 94 °C for 30 s, 50 °C for 30 s, and 72 °C for 7 min; and a final extension at 72 °C for 10 min. Amplicons were separated by electrophoresis through 0.8% agarose gels, purified, cloned into pGEM-T Easy and sequenced by primer-walking as described previously.

### Sequence and phylogenetic analysis

Sequencing data were processed and analysed using CLC MAIN WORKBENCH v. 6.9.2 (QIAGEN) and GENEIOUS v. 11.0.2 (Biomatters) computer software. Sequences were compared to all known badnaviruses on the NCBI database using BLAST algorithms available on the NCBI website (http://blast.ncbi.nlm.nih.gov/Blast.cgi). The presence of putative ORFs was predicted using GENEIOUS v. 11.0.2 and SNAPGENE software (GLS Biotech). Virus sequences were further aligned and analysed with the CLUSTALW multiple alignment application using BIOEDIT v. 7.1.9 sequence alignment editor program (http://www.mbio.ncsu.edu/BioEdit/bioedit.html). Phylogenetic trees were constructed from CLUSTALW-aligned sequences on MEGA v. 7.0 (http://www.megasoftware.net/mega.php), using the maximum-likelihood method and a Kimura 2-parameter model with 1000 bootstrap replications. Pairwise sequence comparison (PASC) was carried out on aligned sequences using GENEIOUS v. 11.0.2 computer software. For taxonomic purposes, the 1.2 kb polymerase gene covering the RT/RNase H domains was used to compare sequences from the different genera in the family *Caulimoviridae* while the core 529 bp sequence of the RT/RNase H-coding region (excluding the BadnaFP/RP primer binding sites) was used to compare sequences from TaBV and TaBCHV isolates.

## Results

### PCR screening and sequence analysis

Of the 392 leaf samples collected from the four countries included in this study, 333 were from taro and 59 were from tannia. Of these, 68 taro samples and 23 tannia samples showed virus-like symptoms including mosaic, feathery mottle, vein clearing, downward curling of leaf blades and stunting. As an initial test for the presence of badnaviruses, TNA was extracted from all samples and PCR carried out using the degenerate BadnaFP/RP primers. An amplicon of the expected size was observed in 70 of 94 samples from Uganda, 54 of 86 samples from Kenya, 25 of 41 samples from Tanzania and 100 of 171 samples from Ethiopia. Of the 392 samples, 223 of 333 taro samples and 26 of 59 tannia samples tested positive, with positive samples identified in all of the 25 districts surveyed in the four countries (Table S1). No consistent symptoms were observed on any of the plants testing positive, with numerous symptomless plants also testing positive.

Ten amplicons from samples collected from each country, which included samples from most districts (Table 1), were randomly selected for further analysis and were subsequently cloned and sequenced. All the samples from Ethiopia, Kenya and Uganda were from taro while from Tanzania, eight samples were from taro and two samples (Tz24 and Tz27) were from tannia. Sequences described in this paper are available in GenBank as accession numbers MG017321–MG017360 and MG833013–MG833014.

Analysis of the sequences from the three clones derived from each isolate revealed 98–99% nucleotide identity. When the consensus sequence of each of the 40 isolates was subjected to a BLAST analysis, 14 isolates showed highest nucleotide identity (96–97%) to a New Caledonian TaBV isolate (AY186614), while the remaining 26 isolates showed highest nucleotide identity (79.1–92.6%) to TaBCHV from China. The Ethiopian isolates showed greatest nucleotide identity to TaBCHV only, while isolates from Tanzania, Uganda and Kenya showed greatest nucleotide identity to either TaBCHV or TaBV. Of the two tannia samples sequenced, Tz24 showed 97% nucleotide identity to TaBV from New Caledonia, whereas Tz27 showed 92% nucleotide identity to TaBCHV from China. Nucleotide sequence identity amongst the 40 East African isolates ranged from 57% to 99%. Within isolates showing greatest nucleotide identity to TaBCHV, nucleotide sequence variability was highest in the 10 Ethiopian isolates, with variability of up to 22.6%. In the other three countries, the nucleotide sequence identity of TaBCHV ranged from 85.2% to 99.9%. For the 14 isolates that were most similar to TaBV, nucleotide sequence identity ranged from 96.5% to 98% across all isolates. The least amount of variability in TaBV was observed between isolates within each country, with the four isolates from Kenya showing 99.2–99.8% nucleotide sequence identity, the five samples from Tanzania showing 97.4–99.9% and the remaining five samples from Uganda showing 98.6–99.8% nucleotide sequence identity.

### RCA and sequence analysis

Following the initial sequence analyses, six isolates showing greatest sequence similarity to TaBV and eight isolates showing greatest sequence similarity to TaBCHV were randomly selected and subjected to RCA in an attempt to amplify the complete genomes. When RCA was carried out on eight isolates with high sequence similarity to TaBCHV, no restriction profiles were observed in any samples following digestion with a range of restriction enzymes that were predicted to cut the full-length published TaBCHV and/or TaBV sequences either once or twice. In contrast, *Stu*I digestion of the RCA product obtained from all six isolates showing highest similarity to TaBV resulted in a single fragment of approximately 8 kb. Further, *Xba*I digestion resulted in three fragments, while no restriction profiles were observed following *Sal*I digestion. Putative full-length *Stu*I digest fragments from the six isolates were cloned and the RT/RNase H-coding region sequenced using primer BadnaFP. Three cloned DNAs for individual isolates generated from RCA were sequenced and showed 99–100% identity. The consensus sequence derived from each RCA-amplified isolate was compared with the consensus PCR-generated sequences described earlier and in all cases the RCA-amplified sequences showed 99–100% nucleotide identity to the PCR-amplified sequences.

**Figure 1:**
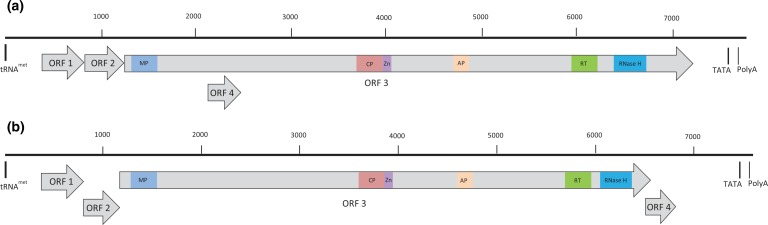
Linearized schematic representation of the genome organization of representative full-length TaBV and TaBCHV sequences from East Africa. (a) Genome organization of full-length TaBV sequences from East Africa representing isolates from Kenya (Ke52), Tanzania (Tz17, Tz24) and Uganda (Ug75). (b) Genome organization of full-length TaBCHV sequences from East Africa representing isolates from Ethiopia (Et17), Kenya (Ke43), Tanzania (Tz27, Tz36) and Uganda (Ug10). The predicted putative conserved domains: MP, movement protein; CP, coat protein; Zn, zinc finger; AP, aspartic protease; RT, reverse transcriptase; RNase H, ribonuclease H are shown on ORF 3. [Colour figure can be viewed at wileyonlinelibrary.com].

Complete genome sequences were then obtained for three representative isolates from taro originating from Kenya (Ke52), Tanzania (Tz17) and Uganda (Ug75), and one isolate infecting tannia from Tanzania (Tz24). The complete genome sequence of isolate Ke52 comprised 7805 nt and contained four ORFs (Fig. 1a; Table S1). ORFs 1–3 were 453, 417 and 5979 nt in length, respectively, and encoded respective putative proteins of 150, 138 and 1992 amino acids (aa). ORF 4 was 333 nt long, encoded a putative protein of 110 aa, and was positioned entirely within ORF 3 (Fig. 1a). The complete genome sequence of isolate Tz17 was 7803 nt with four ORFs similar to Ke52 (Fig. 1a; Table S1). ORFs 1–4of Tz17 were 453, 417, 5982 and 333 nt in length, respectively, and encoded respective putative proteins of 150, 138, 1993 and 110 aa. Similarly, the complete genome of isolate Ug75 was 7796 nt in length and contained four ORFs with a similar arrangement to isolates Ke52 and Tz17 (Fig. 1a). Similar to the TaBV sequences amplified from taro, the complete genome of tannia isolate Tz24 was found to comprise 7799 nt and contain four ORFs (Fig. 1a; Table 1). ORFs 1–4 of Tz24 were 453, 414, 5877 and 330 nt, respectively, which encoded predicted proteins of 150, 137, 1958 and 109 aa, respectively (Fig. 1a).

Sequence analysis of all four genome sequences revealed the presence of a putative tRNA^met^ binding site (5′-TGGTATCAGAGCTTTGTT-3′) with 88% nt identity to the plant tRNA^met^ consensus sequence (3′-ACCAUAGUCUCGGUCCAA-5′). Further, transcriptional promoter elements including a putative TATA box and polyadenylation signal were identified (Table S1). Analysis of the aa sequence of ORF 3 from all four isolates identified conserved motifs related to the MP, CP, AP, RT, RNase H and RNA-binding zinc finger-like domains typical of *Caulimoviridae* (Fig. 1a). Based on these analyses, isolates Ke52, Tz17, Tz24 and Ug75 were identified as TaBV.

### Outward-facing PCR and sequence analysis

Outward-facing PCR was used in an attempt to amplify the complete TaBCHV-like genomic sequence from representative taro samples obtained from Ethiopia (Et17), Kenya (Ke43), Tanzania (Tz36) and Uganda (Ug10) and one tannia sample collected from Tanzania (Tz27). Using sequence-specific primers designed from the consensus RT/RNase H-coding sequences generated previously by PCR, a single amplicon of approximately 7.5 kb was obtained from each isolate. These primers were designed to overlap the BadnaFP/RP amplicons by 202 nt and 163 nt including the primer sequences at the 5′ and 3′ ends, respectively. The amplicons were cloned and complete genome sequences for the five isolates were assembled using the near full-length outward-facing PCR products and the original BadnaFP/RP PCR product sequences. When the overlapping sequences between the two amplicons from each isolate were compared, there was 99–100% identity. The complete genomes of the five isolates varied in length from 7389 to 7654 nt and all contained four putative ORFs (Fig. 1b; Table S1). Whereas the size and arrangement of ORFs 1–3 were similar to that of the TaBCHV isolate from China, putative ORF 4 in all five isolates was located at the 3′ end of ORF 3 where it overlapped the 3′ end of ORF 3 by 77 nt, a position analogous with ORF 5 of the Chinese TaBCHV isolate. In all five isolates, ORFs 1, 2 and 4 comprised 438, 381 and 309 nt, respectively, and encoded putative proteins of 145, 126 and 102 aa, respectively. In contrast, ORF 3 of Et17, Ke43, Tz36, Ug10 and Tz27 comprised 5412, 5388, 5385, 5385 and 5130 nt, respectively, and encoded respective putative proteins of 1803, 1795, 1794, 1794 and 1709 aa (Fig. 1b; Table 1). All five sequences contained the putative tRNA^met^-binding site, which was either 5′-TGGTATCAGAGCTTTGTT-3′ (Et17, Ke43, Tz27 and Ug10) or 5′-TGGTATCA GAGCTTAGTT-3′ (Tz36) and showed 84–88% nucleotide identity to the plant tRNA^met^ consensus sequence. In addition, putative TATA boxes, polyadenylation signals and conserved functional domains typical of *Caulimoviridae* were also identified (Fig. 1b).

### Phylogenetic analysis and pairwise sequence comparison

**Figure 2:**
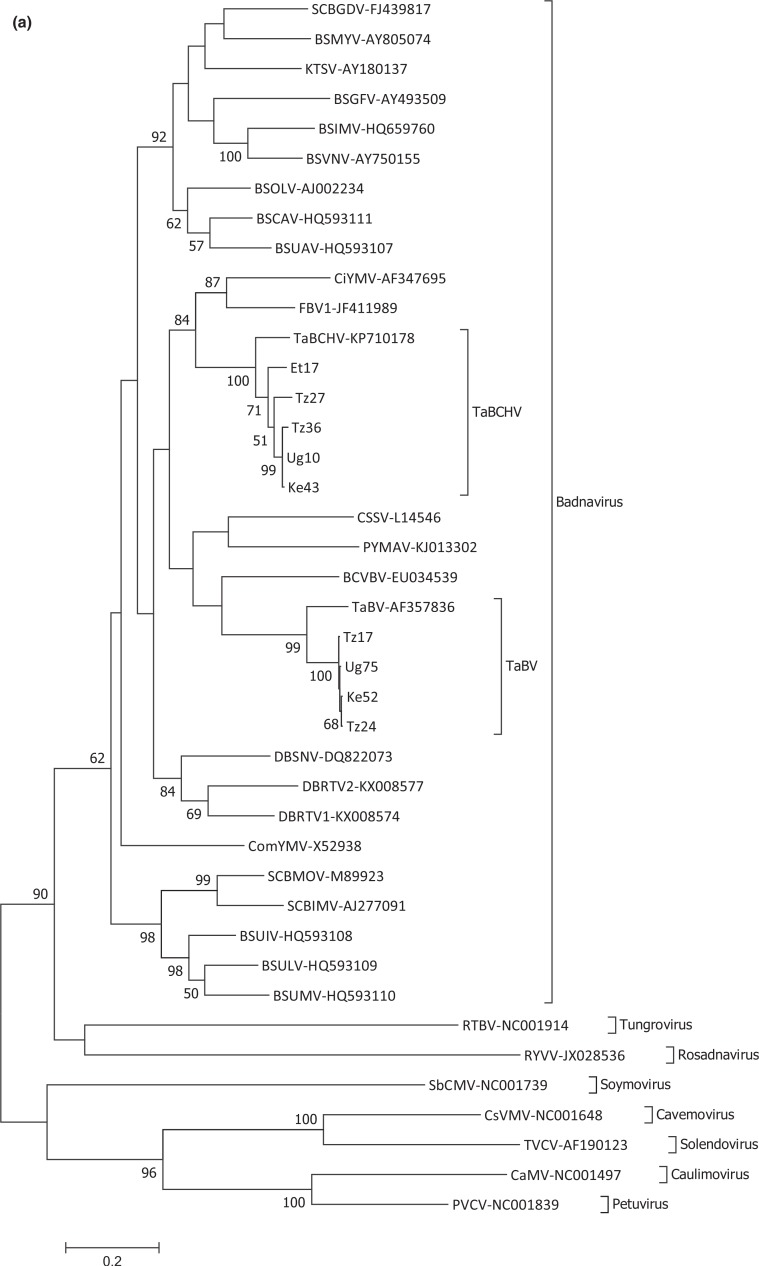

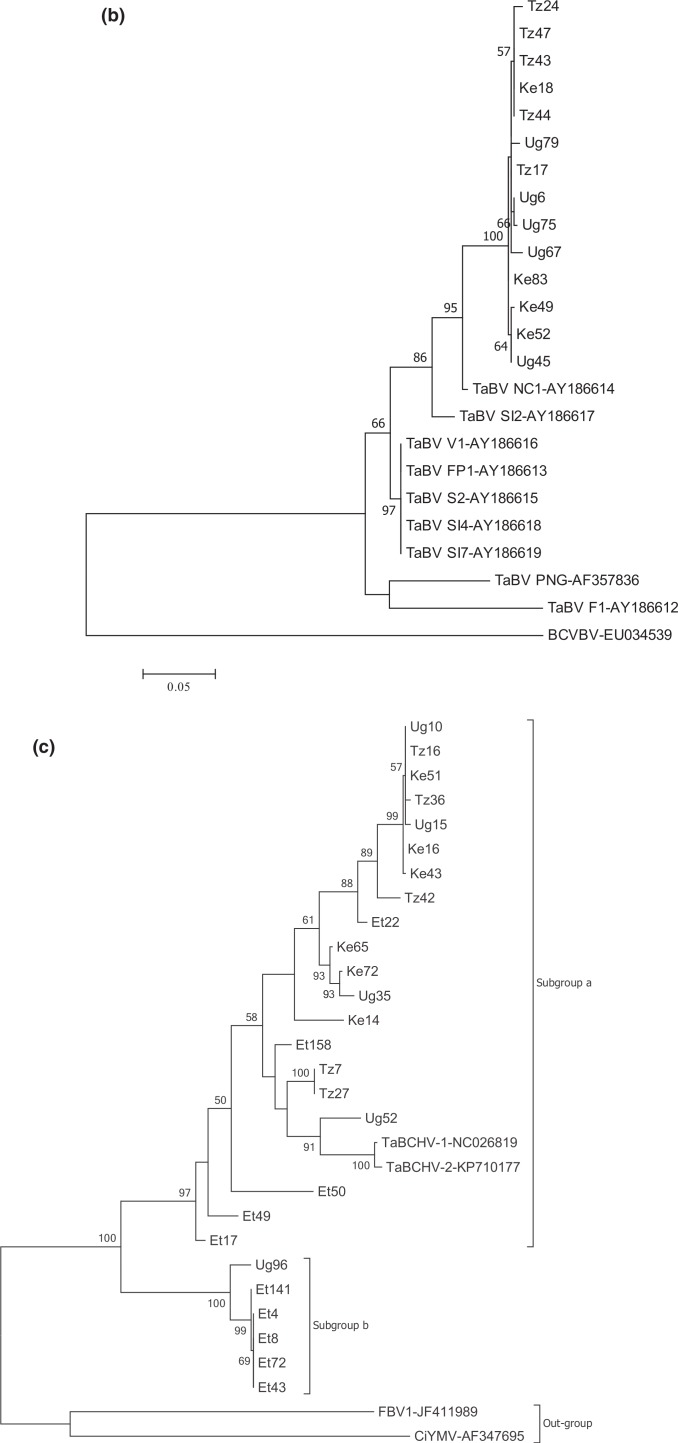
Phylogenetic analyses of the TaBV and TaBCHV sequences characterized in this study. (a) Phylogenetic analysis including representative members of the family *Caulimoviridae* based on 1.2 kb *pol*-gene sequences of the RT/RNase H-coding region of ORF 3 (as described by Geering *et al*., 2010). BSUAV, *Banana streak UA virus*; BSCAV, Banana streak CA virus; BSOLV, *Banana streak OL virus*; BSVNV, *Banana streak VN virus*; BSIMV, *Banana streak IM virus*; KTSV, *Kalanchoe top-spotting virus*; BSGFV, *Banana streak GF virus*; BSMYV, *Banana streak MY virus*; SCBGDV, *Sugarcane bacilliform Guadeloupe D virus*; ComYMV, *Commelina yellow mottle virus*; DBSNV, Dioscorea bacilliform VN virus; DBRTV1, *Dioscorea bacilliform RT virus 1*; DBRTV2, *Dioscorea bacilliform RT virus 2*; FBV1, *Fig badnavirus 1*; CiYMV, *Citrus yellow mosaic virus*; TaBCHV, *Taro bacilliform CH virus*; CSSV, *Cacao swollen shoot virus*; PYMAV, *Pagoda yellow mosaic associated virus*; BCVBV, *Bougainvillea chlorotic vein banding virus*; TaBV, *Taro bacilliform virus*; SCBMOV, *Sugarcane bacilliform MO virus*; SCBIMV, *Sugarcane bacilliform IM virus*; BSUIV, *Banana streak UI virus*; BSULV, *Banana streak UL virus*; BSUMV, *Banana streak UM virus*; RTBV, *Rice tungro bacilliform virus*; CsVMV, *Cassava vein mosaic virus*; TVCV, *Tobacco vein clearing virus*; SbCMV, *Soybean chlorotic mottle virus*; CaMV, *Cauliflower mosaic virus*; PVCV, *Petunia vein clearing virus*; RYVV, *Rose yellow vein virus*. (b) Phylogenetic analysis of TaBV isolates based on core 529 nt RT/RNase H-coding sequences delimited by the BadnaFP/RP primers. Ke, Tz and Ug indicate isolates from Kenya, Tanzania and Uganda, respectively, while TaBV isolates NC1, SI2, V1, FP1, S2, SI4 SI7, PNG and F1 are those previously described by Yang *et al*. (2003b). BCVBV was used as an out-group (a). (c) Phylogenetic analysis of TaBCHV isolates based on core 529 nt RT/RNase H-coding sequences delimited by the BadnaFP/RP primers. Et, Ke, Tz and Ug indicate isolates from Ethiopia, Kenya, Tanzania and Uganda, respectively, while TaBCHV-1 and -2 are described by Kazmi *et al*. (2015). FBV1 and CiYMV were used as out-groups (a).

Phylogenetic analysis was initially carried out using the conserved 1.2 kb RT/RNase H domain sequences of the nine full-length outward-facing PCR- and RCA-generated episomal sequences from this study, together with previously reported TaBV and TaBCHV isolates, additional members of the genus *Badnavirus* and representative members of the other genera in the family *Caulimoviridae*. This analysis confirmed that TaBV and TaBCHV isolates are members of two distinct clades within the genus *Badnavirus* (Fig. 2a). TaBCHV isolates were found to be most closely related to Citrus yellow mosaic virus (AF347695), *Fig badnavirus 1* (JF411989) and several yam-infecting badnavirus species, while TaBV isolates formed a separate clade together with *Bougainvillea chlorotic vein banding virus* (EU034539), *Cacao swollen shoot virus* (L14546) and *Pagoda yellow mosaic associated virus* (KJ013302) (Fig. 2a).

Analysis of full-length and partial TaBV sequences from the 14 isolates from East Africa based on the core 529 bp RT/RNase H sequence showed they were members of a single clade, but they did not form distinct groups based on their country of origin, with isolates from the three countries interspersed across a single terminal branch of the tree (Fig. 2b). The nearest common ancestor to the East African samples was TaBV isolate NC1 from New Caledonia (AY186614).

When analysis was done using the two published TaBCHV sequences from China together with full-length and partial sequences of the 26 isolates from East Africa based on the core 529 bp RT/RNase H sequence, the TaBCHV isolates were divided into two distinct subgroups (Fig. 2c). The first subgroup, herein referred to as ‘subgroup a’, is more diverse and comprises the two published TaBCHV sequences from China together with additional isolates from all four countries in East Africa. The second subgroup, herein referred to as ‘subgroup b’, includes five isolates from Ethiopia and one isolate from Uganda. The distinctive clustering of the six TaBCHV isolates from East Africa (Ug96, Et4, Et8, Et43, Et72 and Et141) within ‘subgroup b’, with high bootstrap support values, is indicative that this subgroup may represent a distinct badnavirus species. ‘Subgroup a’ can be further divided into four closely related sequence groups supported by moderate to high bootstrap values, with three of the Ethiopian TaBCHV isolates in a basal position to these and sharing a common ancestor with ‘sub-group b’.

As the initial sequence comparisons of PCR-amplified RT/RNase H-coding sequences indicated that nucleotide sequence variability in the TaBCHV isolates was up to 22.6%, PASC analysis was carried out using all available TaBCHV sequences (Table S2). This analysis revealed that the six isolates in TaBCHV ‘subgroup b’ showed 79.1–80.5% nucleotide sequence identity with the published TaBCHV sequences from China, which is on the threshold for species demarcation in the genus *Badnavirus*. These six sequences also shared 78.9–81.4% nucleotide sequence identity to other East African TaBCHV isolates, with the exception of two isolates (Et17 and Et49) from ‘subgroup a’ which are distinct from, and basal to, the Chinese TaBCHV sequences with 84.1–85.8% identity, as well as isolate Et22 from another distinct TaBCHV subgroup (Fig. 2c). Five clear sequence groups having very high (>96%) nucleotide sequence identity were identified, including the six isolates from ‘subgroup b’ (96.6–99.9% identity), the two published TaBCHV sequences from China (99.2% identity), isolates Tz7, Tz27 and Et158 (96–100% identity), isolates Ug36, Ke72 and Ke65 (97.9–98.9% identity) and the nine isolates forming the terminal TaBCHV subgroup (96–99.9% identity). Between the various groups of TaBCHV isolates determined in the phylogenetic analysis, nucleotide sequence identity generally ranged from 85% to 94%, which may explain the low bootstrap support for some branches in the phylogenetic analysis (Fig. 2c; Table S2).

## Discussion

Several surveys were carried out in 2014 and 2015 to identify viruses infecting taro and other edible aroids in East Africa. Using a PCR-based strategy with the degenerate badnavirus primers BadnaFP/RP, a high incidence of badnavirus-like sequences was found in taro growing in Ethiopia, Kenya, Tanzania and Uganda. This ranged from 58.4% to 74.4% of samples from each country, with at least one PCR-positive sample detected in every district surveyed. Sequence analysis of the RT/RNase H-coding region of 40 isolates amplified using PCR revealed greatest nucleotide sequence identities to either TaBV or TaBCHV, with 14 samples showing highest (96–97%) nucleotide sequence identity to TaBV from New Caledonia, while the remaining 26 samples showed highest (79–92%) nucleotide sequence identity to TaBCHV from China. In Ethiopia, sequences similar to only TaBCHV were detected, while both TaBV- and TaBCHV-like sequences were detected from Uganda, Kenya and Tanzania. Of the two tannia samples selected for sequencing, TaBV was detected from one sample (Tz24), while TaBCHV was detected from the second sample (Tz27).

Because the BadnaFP/RP-generated amplicons could have been derived from either integrated sequences or episomal virus, RCA was used in an attempt to specifically amplify episomal viral genomic DNA. Whereas RCA amplified the complete genome of TaBV isolates, no amplification products were obtained using samples containing the TaBCHV-like sequences. Therefore, the latter samples were analysed using an outward-facing PCR strategy that resulted in the amplification of full-length East African TaBCHV genomes. Interestingly, analysis of the cloned TaBCHV sequences revealed the presence of the restriction sites *Stu*I and *Xba*I that were predicted from the published TaBCHV sequence from China and which were used to digest the RCA-amplified DNA from these samples. Despite the presence of high molecular weight amplification products in RCA reactions using samples shown to contain TaBCHV, the RCA-amplified products did not digest with *Stu*I and *Xba*I as expected. The reason for this is unknown but could be due to very low levels of target episomal DNA in taro plants, as has been reported with badnaviruses from sweet potato (Kreuze *et al*., 2017).

The genome organization of the TaBV isolates infecting taro from East Africa is consistent with the previously published South Pacific TaBV isolates with four ORFs (Yang *et al*., 2003a). The genome organization of the TaBV isolate infecting tannia is also consistent with the taro-infecting TaBV isolates identified from East Africa and the South Pacific. In contrast, whereas the genome organization of the four TaBCHV isolates from East Africa were similar to each other and also contained four ORFs, this differs from the previously published Chinese TaBCHV isolate, which was reported to encode six ORFs (Kazmi *et al*., 2015). Recently, Wang *et al*. (2018) reported a full-length sequence of TaBCHV infecting taro from Hawaii, USA. The genome of this Hawaiian TaBCHV isolate contained five ORFs. The sizes and locations of ORF 1, 2, 3 and 5 are consistent with ORFs 1–4 of TaBCHV isolates from East Africa. However, unlike TaBCHV isolates from East Africa, TaBCHV-Hawaii possesses an overlapping ORF within ORF 3 (Wang *et al*., 2018). Of the five East African TaBCHV isolates sequenced in the current study, three (Ke43, Ug10 and Tz36) are representative of a small subset in the terminal branch of ‘subgroup a’ in the phylogenetic analysis, while Et17 is a basal member of this subgroup. The sole TaBCHV isolate from tannia (Tz27) formed another small subset within ‘subgroup a’ together with previously published TaBCHV isolates from China and other isolates from Ethiopia and Uganda. Based on the genome organization and phylogenetic analysis, it could be inferred that all members of ‘subgroup a’ would have four ORFs, but interestingly the Chinese TaBCHV sequence, which falls into a distinct group of isolates within ‘subgroup a’, has two additional ORFs. One of these ORFs is analogous to the TaBV ORF4, while the other, ORF 6, is located at a position downstream of the ORF4 described herein from TaBCHV isolates from East Africa. Additional sequencing of isolates from the various TaBCHV groups within ‘sub-group a’ of the phylogenetic tree is needed to clarify these differences in genome organization.

Phylogenetic analysis showed that all East African TaBV isolates form a single subgroup within known TaBV isolates and are most similar to a published isolate from New Caledonia. This may indicate that a single isolate of TaBV was initially introduced to East Africa and has since been disseminated throughout three of the countries in the region. Phylogenetic analysis of TaBCHV isolates from East Africa showed that they form two distinct subgroups. PASC of the isolates within these two subgroups suggests that ‘subgroup b’ may be distinct enough from some members of ‘subgroup a’ to be considered a distinct species. However, when all sequences in this group are considered there is no clear delineation of species based on the current criteria for species demarcation in the genus *Badnavirus* of 20% nucleotide sequence variability in the core RT/RNase H-coding region of ORF3 (Table S2). Whether the members of ‘subgroup b’ represent a novel badnavirus species requires further sequencing of TaBCHV isolates from East Africa and other regions, and this will be the focus of future research.

Virus infection in taro has been reported to affect both the quality and quantity of the harvested corms, with production losses ranging from 20% to 60% and, in some cases, plant death. These losses often result from the synergistic interactions of multiple virus infections (Rana *et al*., 1983; Elliott *et al*., 1997; Revill *et al*., 2005); however, the role of badnaviruses in these interactions remains poorly understood. Similar to previous studies (Yang *et al*., 2003b; Revill *et al*., 2005), no correlation was observed between the presence of the badnavirus-like sequences and symptoms in either taro or tannia plants in this study, with virus sequences amplified from plants both with and without symptoms using PCR and RCA. However, because mixed infections are common in taro (Revill *et al*., 2005), testing the samples for other viruses is necessary to shed further light on any symptoms associated with badnavirus infection. In summary, this study confirmed the widespread occurrence of two known badnavirus species, TaBV and TaBCHV, in East Africa. Furthermore, in the case of TaBCHV, at least two genetically distinct subgroups were identified. To the authors’ knowledge, this is the first report of TaBV and TaBCHV in these countries and the first sequence record from tannia.

## Supporting Information

**Table S1.** Summary of the genomic features of TaBV and TaBCHV isolates from East Africa.

**Table S2.** Pairwise sequence comparisons of TaBCHV isolates using core 529 nt RT/RNase H-coding sequences delimited by the BadnaFP/RP primers.
