# Supplementary material for: Identification and molecular characterization of Taro bacilliform virus and Taro bacilliform CH virus from East Africa
Source: Plant Pathol. 2018 Aug 31;67:1977–86. doi: 10.1111/ppa.12921 (PMC7198128; doi:10.1111/ppa.12921)
Supplement: Supplementary file 1 [file PPA-2018-PPA-12921-s1.docx]

| Virus species | Isolate | Genome  length (nt) | ORF 1 | | | | ORF 2 | | | | ORF 3 | | | | ORF 4 | | | | Transcriptional elements | | |
| --- | --- | --- | --- | --- | --- | --- | --- | --- | --- | --- | --- | --- | --- | --- | --- | --- | --- | --- | --- | --- | --- |
|  |  |  | nt | Start-stop | aa | Protein MW | nt | Start-stop | aa | Protein MW | nt | Start-stop | aa | Protein MW | nt | Start-stop | aa | Protein MW | TATA box | -gap- | polyA-signal |
|  |  |  | length | (codon use) | length | (kDa) | length | (codon use) | length | (kDa) | length | (codon use) | length | (kDa) | length | (codon use) | length | (kDa) |  |  |  |
| TaBV | Ke52 | 7805 | 453 | 386-838 | 150 | 17.1 | 417 | 838-1254 | 138 | 15.1 | 5979 | 1257-7235 | 1992 | 227 | 333 | 2137-2469 | 110 | 12.5 | 7609-7615 | -99- | 7714-7720 |
|  |  |  |  | (ATG-TGA) |  |  |  | (ATG-TAA) |  |  |  | ATG-TAA |  |  |  | (ATG-TAA) |  |  | ttcTATAAAAggc |  | TTTTTT |
|  | Tz17 | 7803 | 453 | 386-838 | 150 | 17.1 | 417 | 838-1254 | 138 | 15.1 | 5982 | 1257-7238 | 1993 | 227.1 | 333 | 2137-2469 | 110 | 12.8 | 7612-7618 | -94- | 7713-7718 |
|  |  |  |  | (ATG-TGA) |  |  |  | (ATG-TAA) |  |  |  | (ATG-TAA) |  |  |  | (ATG-TAA) |  |  | tccTATAAAAggc |  | TTTATT |
|  | Ug75 | 7796 | 453 | 386-838 | 150 | 17.1 | 414 | 838-1251 | 137 | 15 | 5976 | 1254-7229 | 1991 | 226.8 | 327 | 2134-2460 | 108 | 12.5 | 7603-7609 | -98- | 7708-7713 |
|  |  |  |  | (ATG-TGA) |  |  |  | (ATG-TAA) |  |  |  | (ATG-TAA) |  |  |  | (ATG-TAA) |  |  | ttcTATAAAAggc |  | TTTTTT |
|  | Tz24 | 7799 | 453 | (ATG-TGA) | 150 | 17.1 | 414 | 838-1251 | 137 | 15 | 5877 | 1251-7127 | 1958 | 222.7 | 330 | 2131-2460 | 109 | 12.6 | 7605-7611 | -97- | 7709-7714 |
|  |  |  |  | (386-838) |  |  |  | (ATG-TAA) |  |  |  | (ATG-TAA) |  |  |  | (ATG-TAA) |  |  | ttcTATAAAAggc |  | TTTTTT |
| TaBCHV | Et17 | 7610 | 438 | 359-796 | 145 | 17 | 381 | 793-1173 | 126 | 14 | 5412 | 1170-6581 | 1803 | 205.9 | 309 | 6502-6810 | 102 | 12.2 | 7561-7467 | -94- | 7562-7567 |
|  |  |  |  | (ATG-TGA) |  |  |  | (ATG-TGA) |  |  |  | (ATG-TGA) |  |  |  | (ATG-TGA) |  |  | aggTATATAAtaa |  | AAAAAT |
|  | Ke43 | 7647 | 438 | 344-781 | 145 | 17 | 381 | 778-1158 | 126 | 13.9 | 5388 | 1163-6550 | 1795 | 200.4 | 309 | 6471-6779 | 102 | 12.4 | 7490-7496 | -95- | 7592-7597 |
|  |  |  |  | (ATG-TGA) |  |  |  | (ATG-TGA) |  |  |  | (ATG-TGA) |  |  |  | (ATG-TGA) |  |  | aggTATATAAtat |  | AAAAAT |
|  | Tz36 | 7654 | 438 | 521-958 | 145 | 16.7 | 381 | 955-1335 | 126 | 14 | 5385 | 1341-6725 | 1794 | 203.8 | 309 | 6646-6954 | 102 | 12.4 | 7425-7431 | -112- | 7544-7549 |
|  |  |  |  | (ATG-TGA) |  |  |  | (ATG-TGA) |  |  |  | (ATG-TGA) |  |  |  | (ATG-TGA) |  |  | atcTATATAAgga |  | TAAAAA |
|  | Ug10 | 7643 | 438 | 344-781 | 145 | 17 | 381 | 778-1158 | 126 | 14 | 5385 | 1163-6547 | 1794 | 206.3 | 309 | 6468-6776 | 102 | 12.4 | 7244-7250 | -112- | 7363-7368 |
|  |  |  |  | (ATG-TGA) |  |  |  | (ATG-TGA) |  |  |  | (ATG-TGA) |  |  |  | (ATG-TGA) |  |  | atcTATATAAgga |  | TAAAAA |
|  | Tz27 | 7389 | 438 | 344-781 | 145 | 17 | 381 | 778-1185 | 126 | 14 | 5130 | 1164-6292 | 1709 | 193.8 | 309 | 6214-6522 | 102 | 12.4 | 6990-6996 | -112- | 7109-7114 |
|  |  |  |  | (ATG-TGA) |  |  |  | (ATG-TGA) |  |  |  | (ATG-TGA) |  |  |  | (ATG-TGA) |  |  | atcTATATAAgga |  | TAAAAA |
| nt: nucleotide; aa: amino acid; MW: molecular weight; kDa: kilodalton | | | | | | | | | | | | | | | | | | | | | |

**Table S1:** Summary of the genomic features of TaBV and TaBCHV isolates from East Africa.
