# Supplementary material for: Identification and molecular characterization of Taro bacilliform virus and Taro bacilliform CH virus from East Africa
Source: Plant Pathol. 2018 Aug 31;67:1977–86. doi: 10.1111/ppa.12921 (PMC7198128; doi:10.1111/ppa.12921)
Supplement: Supplementary file 2 [file PPA-2018-PPA-12921-s2.docx]

**Table S2:** Pairwise sequence comparisons of TaBCHV isolates using core 529 nt RT/RNase H-coding sequences delimited by the BadnaFP/RP primers.

|  | Tz16 | Ke51 | Ug10 | Tz36 | Ug15 | Ke16 | Ke43 | Tz42 | Et22 | Ug35 | Ke72 | Ke65 | Ke14 | TaBCHV-1 | TaBCHV-2 | Ug52 | Tz27 | Tz7 | Et158 | Et49 | Et17 | Et50 | Et8 | Et4 | Et43 | Et72 | Et141 |
| --- | --- | --- | --- | --- | --- | --- | --- | --- | --- | --- | --- | --- | --- | --- | --- | --- | --- | --- | --- | --- | --- | --- | --- | --- | --- | --- | --- |
| Tz16 |  |  |  |  |  |  |  |  |  |  |  |  |  |  |  |  |  |  |  |  |  |  |  |  |  |  |  |
| Ke51 | 99.9 |  |  |  |  |  |  |  |  |  |  |  |  |  |  |  |  |  |  |  |  |  |  |  |  |  |  |
| Ug10 | 99.9 | 99.9 |  |  |  |  |  |  |  |  |  |  |  |  |  |  |  |  |  |  |  |  |  |  |  |  |  |
| Tz36 | 99.6 | 99.6 | 99.6 |  |  |  |  |  |  |  |  |  |  |  |  |  |  |  |  |  |  |  |  |  |  |  |  |
| Ug15 | 99.6 | 99.6 | 99.6 | 99.2 |  |  |  |  |  |  |  |  |  |  |  |  |  |  |  |  |  |  |  |  |  |  |  |
| Ke16 | 99.8 | 99.8 | 99.8 | 99.4 | 99.4 |  |  |  |  |  |  |  |  |  |  |  |  |  |  |  |  |  |  |  |  |  |  |
| Ke43 | 99.6 | 99.6 | 99.6 | 99.2 | 99.2 | 99.8 |  |  |  |  |  |  |  |  |  |  |  |  |  |  |  |  |  |  |  |  |  |
| Tz42 | 96.0 | 96.0 | 96.0 | 95.6 | 95.6 | 96.2 | 96.4 |  |  |  |  |  |  |  |  |  |  |  |  |  |  |  |  |  |  |  |  |
| Et22 | 96.4 | 96.4 | 96.4 | 96.0 | 96.0 | 96.2 | 96.0 | 96.0 |  |  |  |  |  |  |  |  |  |  |  |  |  |  |  |  |  |  |  |
| Ug35 | 91.3 | 91.3 | 91.3 | 90.9 | 91.3 | 91.1 | 90.9 | 93.4 | 94.1 |  |  |  |  |  |  |  |  |  |  |  |  |  |  |  |  |  |  |
| Ke72 | 91.8 | 91.8 | 91.8 | 91.5 | 91.8 | 92.0 | 91.8 | 93.9 | 94.7 | 98.7 |  |  |  |  |  |  |  |  |  |  |  |  |  |  |  |  |  |
| Ke65 | 92.6 | 92.6 | 92.6 | 92.2 | 92.6 | 92.8 | 92.6 | 94.7 | 95.4 | 97.9 | 98.9 |  |  |  |  |  |  |  |  |  |  |  |  |  |  |  |  |
| Ke14 | 89.2 | 89.2 | 89.2 | 88.8 | 89.2 | 89.4 | 89.2 | 89.9 | 91.3 | 92.6 | 93.4 | 93.7 |  |  |  |  |  |  |  |  |  |  |  |  |  |  |  |
| TaBCHV-1 | 87.3 | 87.3 | 87.3 | 86.9 | 87.7 | 87.1 | 86.9 | 86.1 | 88.0 | 89.0 | 88.8 | 89.2 | 90.3 |  |  |  |  |  |  |  |  |  |  |  |  |  |  |
| TaBCHV-2 | 86.9 | 86.9 | 86.9 | 86.5 | 87.3 | 86.7 | 86.5 | 85.8 | 87.7 | 88.6 | 88.4 | 88.8 | 89.9 | 99.2 |  |  |  |  |  |  |  |  |  |  |  |  |  |
| Ug52 | 87.9 | 87.9 | 87.9 | 87.5 | 88.2 | 87.7 | 87.5 | 86.7 | 88.4 | 88.6 | 89.0 | 89.0 | 90.5 | 92.6 | 92.2 |  |  |  |  |  |  |  |  |  |  |  |  |
| Tz27 | 91.8 | 91.8 | 91.8 | 91.5 | 91.8 | 91.7 | 91.5 | 90.3 | 92.0 | 91.7 | 92.0 | 92.4 | 93.2 | 91.5 | 91.1 | 93.0 |  |  |  |  |  |  |  |  |  |  |  |
| Tz7 | 91.8 | 91.8 | 91.8 | 91.5 | 91.8 | 91.7 | 91.5 | 90.3 | 92.0 | 91.7 | 92.0 | 92.4 | 93.2 | 91.5 | 91.1 | 93.0 | 99.9 |  |  |  |  |  |  |  |  |  |  |
| Et158 | 92.4 | 92.4 | 92.4 | 92.0 | 92.0 | 92.2 | 92.0 | 90.5 | 92.6 | 92.0 | 92.4 | 92.8 | 91.8 | 92.0 | 91.7 | 92.6 | 96.0 | 96.0 |  |  |  |  |  |  |  |  |  |
| Et49 | 89.4 | 89.4 | 89.4 | 89.0 | 89.4 | 89.6 | 89.4 | 89.4 | 91.5 | 89.8 | 90.5 | 90.7 | 89.8 | 88.8 | 88.4 | 88.8 | 90.9 | 90.9 | 92.2 |  |  |  |  |  |  |  |  |
| Et17 | 90.5 | 90.5 | 90.5 | 90.1 | 90.5 | 90.3 | 90.1 | 90.9 | 93.7 | 91.8 | 92.2 | 92.4 | 90.3 | 88.6 | 88.2 | 88.4 | 91.7 | 91.7 | 93.4 | 96.0 |  |  |  |  |  |  |  |
| Et50 | 86.3 | 86.3 | 86.3 | 86.0 | 86.3 | 86.1 | 86.0 | 85.2 | 86.9 | 87.3 | 87.1 | 87.1 | 86.3 | 89.0 | 88.6 | 86.7 | 89.2 | 89.2 | 91.5 | 91.1 | 91.1 |  |  |  |  |  |  |
| Et8 | 81.0 | 81.0 | 81.0 | 80.6 | 80.8 | 80.8 | 80.6 | 81.2 | 84.1 | 79.5 | 79.9 | 80.6 | 80.8 | 79.7 | 79.1 | 78.9 | 80.1 | 80.1 | 80.5 | 84.1 | 85.8 | 77.6 |  |  |  |  |  |
| Et4 | 81.0 | 81.0 | 81.0 | 80.6 | 80.8 | 80.8 | 80.6 | 81.2 | 84.1 | 79.5 | 79.9 | 80.6 | 80.8 | 79.7 | 79.1 | 78.9 | 80.1 | 80.1 | 80.5 | 84.1 | 85.8 | 77.6 | 99.9 |  |  |  |  |
| Et43 | 81.0 | 81.0 | 81.0 | 80.6 | 80.8 | 80.8 | 80.6 | 81.2 | 84.1 | 79.5 | 79.9 | 80.6 | 80.8 | 79.7 | 79.1 | 78.9 | 80.1 | 80.1 | 80.5 | 84.1 | 85.8 | 77.6 | 99.9 | 99.9 |  |  |  |
| Et72 | 81.0 | 81.0 | 81.0 | 80.6 | 80.8 | 80.8 | 80.6 | 81.2 | 84.1 | 79.5 | 79.9 | 80.6 | 80.8 | 79.7 | 79.1 | 78.9 | 80.1 | 80.1 | 80.5 | 84.1 | 85.8 | 77.6 | 99.9 | 99.9 | 99.9 |  |  |
| Et141 | 81.2 | 81.2 | 81.2 | 80.8 | 81.0 | 81.0 | 80.8 | 81.4 | 84.3 | 79.7 | 80.1 | 80.8 | 81.0 | 79.9 | 79.3 | 79.1 | 80.3 | 80.3 | 80.6 | 84.3 | 85.6 | 77.4 | 99.8 | 99.8 | 99.8 | 99.8 |  |
| Ug96 | 81.2 | 81.2 | 81.2 | 80.8 | 81.0 | 81.0 | 80.8 | 81.0 | 84.3 | 79.5 | 79.9 | 80.6 | 81.6 | 80.5 | 79.9 | 79.7 | 80.5 | 80.5 | 80.8 | 84.3 | 85.2 | 77.6 | 96.6 | 96.6 | 96.6 | 96.6 | 96.8 |

TaBCHV-1 is GenBank Accession No. NC026819; TaBCHV-2 is GenBank Accession No. KP710177
